# PLC-γ-Ca^2+^ pathway regulates axonal TrkB endocytosis and is required for long-distance propagation of BDNF signaling

**DOI:** 10.3389/fnmol.2024.1009404

**Published:** 2024-04-10

**Authors:** Guillermo Moya-Alvarado, Xavier Valero-Peña, Alejandro Aguirre-Soto, Fernando J. Bustos, Oscar M. Lazo, Francisca C. Bronfman

**Affiliations:** ^1^Faculty of Biological Sciences, Pontificia Universidad Catolica de Chile (UC), Santiago, Chile; ^2^NeuroSignaling Laboratory, Institute of Biomedical Sciences (ICB), Faculty of Medicine and Faculty of Life Sciences, Universidad Andres Bello, Santiago, Chile; ^3^Constantin-Paton Research Laboratory, Institute of Biomedical Sciences (ICB), Faculty of Medicine and Faculty of Life Sciences, Universidad Andres Bello, Santiago, Chile; ^4^Department of Neuromuscular Diseases, UCL Queen Square Institute of Neurology, University College London, London, United Kingdom

**Keywords:** PLC-γ, brain-derived neurotrophic factor, TrkB, axonal transport, signaling endosomes, dendritic branching, calcium, endocytosis

## Abstract

Brain-derived neurotrophic factor (BDNF) and its tropomyosin receptor kinase B (TrkB) are important signaling proteins that regulate dendritic growth and maintenance in the central nervous system (CNS). After binding of BDNF, TrkB is endocytosed into endosomes and continues signaling within the cell soma, dendrites, and axon. In previous studies, we showed that BDNF signaling initiated in axons triggers long-distance signaling, inducing dendritic arborization in a CREB-dependent manner in cell bodies, processes that depend on axonal dynein and TrkB activities. The binding of BDNF to TrkB triggers the activation of different signaling pathways, including the ERK, PLC-γ and PI3K-mTOR pathways, to induce dendritic growth and synaptic plasticity. How TrkB downstream pathways regulate long-distance signaling is unclear. Here, we studied the role of PLC-γ-Ca^2+^ in BDNF-induced long-distance signaling using compartmentalized microfluidic cultures. We found that dendritic branching and CREB phosphorylation induced by axonal BDNF stimulation require the activation of PLC-γ in the axons of cortical neurons. Locally, in axons, BDNF increases PLC-γ phosphorylation and induces intracellular Ca^2+^ waves in a PLC-γ-dependent manner. In parallel, we observed that BDNF-containing signaling endosomes transport to the cell body was dependent on PLC-γ activity and intracellular Ca^2+^ stores. Furthermore, the activity of PLC-γ is required for BDNF-dependent TrkB endocytosis, suggesting a role for the TrkB/PLC-γ signaling pathway in axonal signaling endosome formation.

## Introduction

Brain-derived neurotrophic factor (BDNF) and its receptor TrkB are major regulators of dendritic branching in cortical and hippocampal neurons (Xu et al., 2000; Horch and Katz, 2002; Cheung et al., 2007). TrkB is located both in the somatodendritic compartment and axonal compartment (AC) of neurons (Arimura et al., 2009), and BDNF is released in both dendrites and axons (Matsuda et al., 2009). Upon binding to TrkB, BDNF activates three main downstream signaling pathways, phospholipase C- γ (PLC-γ), extracellular signal-regulated kinases (ERKs), and phosphoinositide 3-kinase (PI3K) and mechanistic Target of Rapamycin protein kinase (mTOR) (Reichardt, 2006; Saxton and Sabatini, 2017), which regulate dendritic and axonal growth (Huang and Reichardt, 2003; Gonzalez et al., 2016), synaptic transmission (Kang and Schuman, 1995; Zhang et al., 2013) and learning and memory (Alonso et al., 2002a,b; Egan et al., 2003). Extracellular application of BDNF induces an increase in cytoplasmic Ca^2+^ in a PLC-γ-dependent manner (Li et al., 2005). Furthermore, it has been shown that Ca^2+^ influx increases dendritic arborization by triggering a transcriptional program that involves the activation of calmodulin kinase IV (CAMKIV) and phosphorylation of the cAMP response element-binding protein (CREB) transcription factor (Redmond et al., 2002). BDNF, in turn, promotes the release of intracellular Ca^2+^ stores by activation of the inositol 3,4,5-triphosphate receptor (IP3R), as well as the entry of extracellular Ca^2+^ by opening transient receptor-potential cation channel subfamily C (TRPC) (Li et al., 2005; Leal et al., 2015). In line with this evidence, BDNF-dependent long-term potentiation in the hippocampus requires both pre- and post-synaptic PLC-γ activity (Gärtner et al., 2006; Gruart et al., 2007).

In cortical and hippocampal neurons, the binding of BDNF to TrkB promotes its dimerization and autophosphorylation, inducing the internalization of the receptor into signaling endosomes (Bronfman et al., 2014; Cosker and Segal, 2014; Scott-Solomon and Kuruvilla, 2018; Moya-Alvarado et al., 2022). We have previously shown that BDNF axonal signaling promotes dendritic arborization in a TrkB-dependent manner (Moya-Alvarado et al., 2023). TrkB is endocytosed in axons and retrogradely transported to the cell body by the molecular motor dynein. In the cell body, activated TrkB upregulates CREB, increasing the transcription and translation of proteins (Moya-Alvarado et al., 2023). Although long-distance signaling of BDNF has been described in cortical neurons (Cohen et al., 2011; Zhou et al., 2012), little is known about the cellular mechanism and the downstream signaling pathways regulating this process. Several lines of evidence support the participation of the PLC-γ and increased intracellular Ca^2+^ in signaling endosome regulation and long-distance signaling mediated by BDNF. It has been reported that in hippocampal neurons, the internalization of the BDNF/TrkB complex is regulated by an increase in Ca^2+^ influx through N-methyl-d-aspartate receptors (NMDARs) (Du et al., 2003). Furthermore, the axonal stimulation of retinal ganglionic cells with BDNF promotes a retrograde potentiation of retinal synapses in a TrkB- and PLC-γ-dependent manner (Lom et al., 2002; Du and Poo, 2004), suggesting that PLC-γ participate in BDNF long-distance signaling. Interestingly, in the axons of sympathetic neurons, TrkA via PLC-γ regulates the activation of calcineurin to dephosphorylate dynamin 1, coordinating receptor endocytosis and axonal growth (Bodmer et al., 2011). Taken together, these results suggest that activation of the PLC-γ-Ca^2+^ pathway participates in the endocytosis and trafficking of Trks receptors.

In previous studies, using compartmentalized cultures of cortical neurons, we demonstrated that PI3K activity is not necessary for the internalization or transport of BDNF/TrkB signaling endosomes. Instead, it plays a role in regulating mTOR-dependent translation of CREB-target genes in cell bodies (Moya-Alvarado et al., 2023). Here, we show that axonal PLC-γ activity and/or Ca^2+^signaling were required for BDNF-induced dendritic branching, CREB phosphorylation, and the accumulation of BDNF-labeled endosomes in cell bodies. As we explored the levels at which this regulation occurs, we discovered that the effect is primarily at the level of axonal signaling, rather than at the cell body, as it was the case for PI3K activity. Consistent with previous findings, our work reveals that BDNF increases axonal Ca^2+^ levels in a PLC-γ-dependent manner (Du and Poo, 2004). Moreover, BDNF triggers the activation of PLC-γ in axons. To delve deeper into whether the regulation occurs at the levels of endocytosis or transport, we initially investigated endocytosis since it precedes transport. Finally, we show that the PLC-γ-Ca^2+^ signaling pathway is required for endocytosis of TrkB, providing a mechanism by which axonal PLC-γ regulate the formation of signaling endosomes for retrograde BDNF/TrkB signaling.

## Results

### Axonal PLC-γ regulates BDNF/TrkB long-distance signaling in cortical neurons

**Figure 1:**
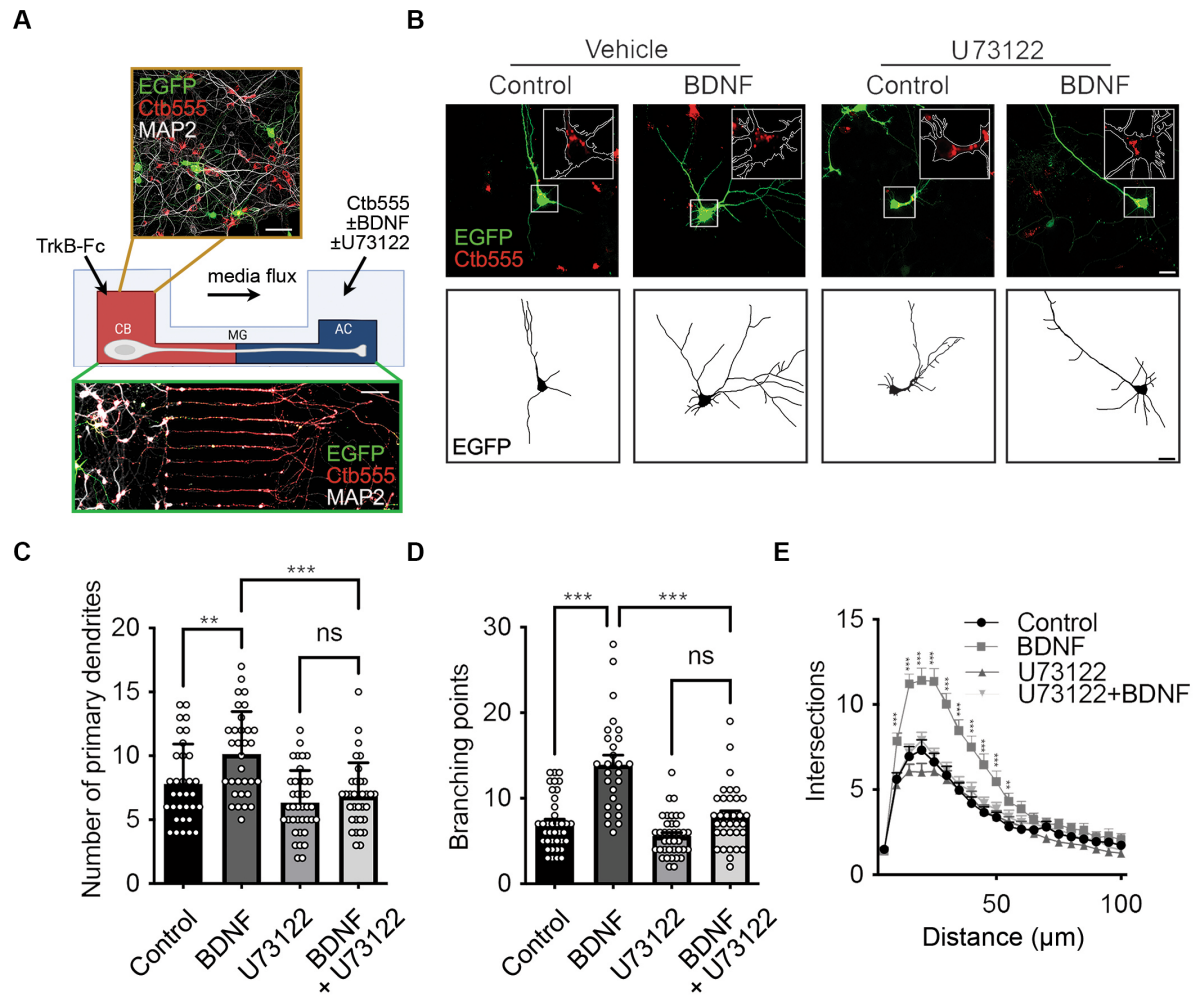
Axonal PLC-γ activity is required for dendritic arborization induced by BDNF in rat cortical neurons. **(A)** Schematics showing the experimental design and example of a lower and a higher magnification image of the cortical neurons in compartmentalized culture. Cortical neurons (DIV 6) were transfected with a plasmid expressing EGFP (green). The cell body compartment (CB) was incubated with TrkB-Fc (100 ng/ml). The axonal compartment (AC) was stimulated with BDNF (50 ng/ml) in addition to Ctb555 (red) in the presence or absence of the PLC-γ inhibitor U73122 (5 μM). Treatments were applied for 48 h. Finally, neurons were fixed, and immunofluorescence was performed against MAP2 (white). Upper panel, scale bar 50 μm. Lower panel, scale bar 50 μm. **(B)** Upper panels, representative images of the CB of compartmentalized rat cortical neurons whose axons were treated with DMSO (control), U73122, BDNF or BDNF following preincubation with U73122. Ctb555 is shown in red and EGFP in green. Insets are 2.5x-zoom images of the Ctb555 signal and the cell body contour is labeled with a white line. Lower panels, binary images obtained from EGFP-associated fluorescence was used to perform morphological analysis. Scale bar, 20 μm. **(C–E)** Quantification of primary dendrites **(C)** and branching points **(D)** and Sholl analysis **(E)** for neurons labeled with EGFP/MAP2/Ctb555 for each treatment. *n* = 27–38 neurons from 3 independent experiments. The results are expressed as the means ± SEMs. **p < 0.01, ***p < 0.001. Statistical analysis was performed by one-way ANOVA followed by the Bonferroni correction for multiple comparisons **(C,D)**. The Sholl’s analysis results were statistically analyzed by two-way ANOVA followed by the Bonferroni correction for multiple comparisons **(E)**.

We have previously reported that axonal stimulation with BDNF promotes dendritic arborization in a CREB-dependent manner. Interestingly, when characterizing the downstream TrkB axonal signaling pathways, we found that PI3K activity is required in the cell bodies but not in axons of cortical neurons to induce dendritic arborization (Moya-Alvarado et al., 2023). One downstream pathway involved in TrkB signaling in axons is PLC-γ, as previously shown for RGCs and sympathetic neurons (Du and Poo, 2004; Bodmer et al., 2011). Using our previously described *in vitro* model of compartmentalized cultures of cortical neurons (Moya-Alvarado et al., 2023), we evaluated the changes in dendritic morphology induced by axonal stimulation with BDNF and investigated whether treating axons with a well-known pharmacological aminosteroid PLC-γ antagonist, U73122 (Bleasdale et al., 1990; Smith et al., 1990; Smallridge et al., 1992), affects the retrograde signaling of BDNF. In brief, we expressed EGFP in DIV 6 cortical neurons plated in microfluidic devices with 300 μm long microgrooves (Moya-Alvarado et al., 2024). We added a TrkB-Fc chimeric protein in the cell body compartment (CB) to neutralize the activity of endogenous BDNF released by neurons (Shelton et al., 1995). Notice that the media flux is toward the axons; therefore, the cell body compartment is fluidically isolated from the axonal compartment, as indicated in Figure 1A. To identify neurons projecting their axons to the axonal compartment (AC), we used a fluorescently labeled subunit B of cholera toxin (Ctb) (Escudero et al., 2019) that is internalized in axons and retrogradely transported up to the Golgi apparatus of neurons that have axons in the AC (Figures 1A,B). To identify the somatodendritic domain of neurons, MAP2 immunofluorescence was performed and EGFP fluorescence was used to study arborization. Similar to what we have previously observed, the addition of BDNF to axons increased the number of primary dendrites (Figures 1B,C), the number of branching points (Figures 1B,D), as well as the arborization (Figure 1E) in rat cortical neurons (Moya-Alvarado et al., 2023). However, the addition of U73122 in the AC prevented these effects of BDNF (Figures 1B–E), suggesting that PLC-γ is required for the long-distance axonal signaling of BDNF.

**Figure 2:**
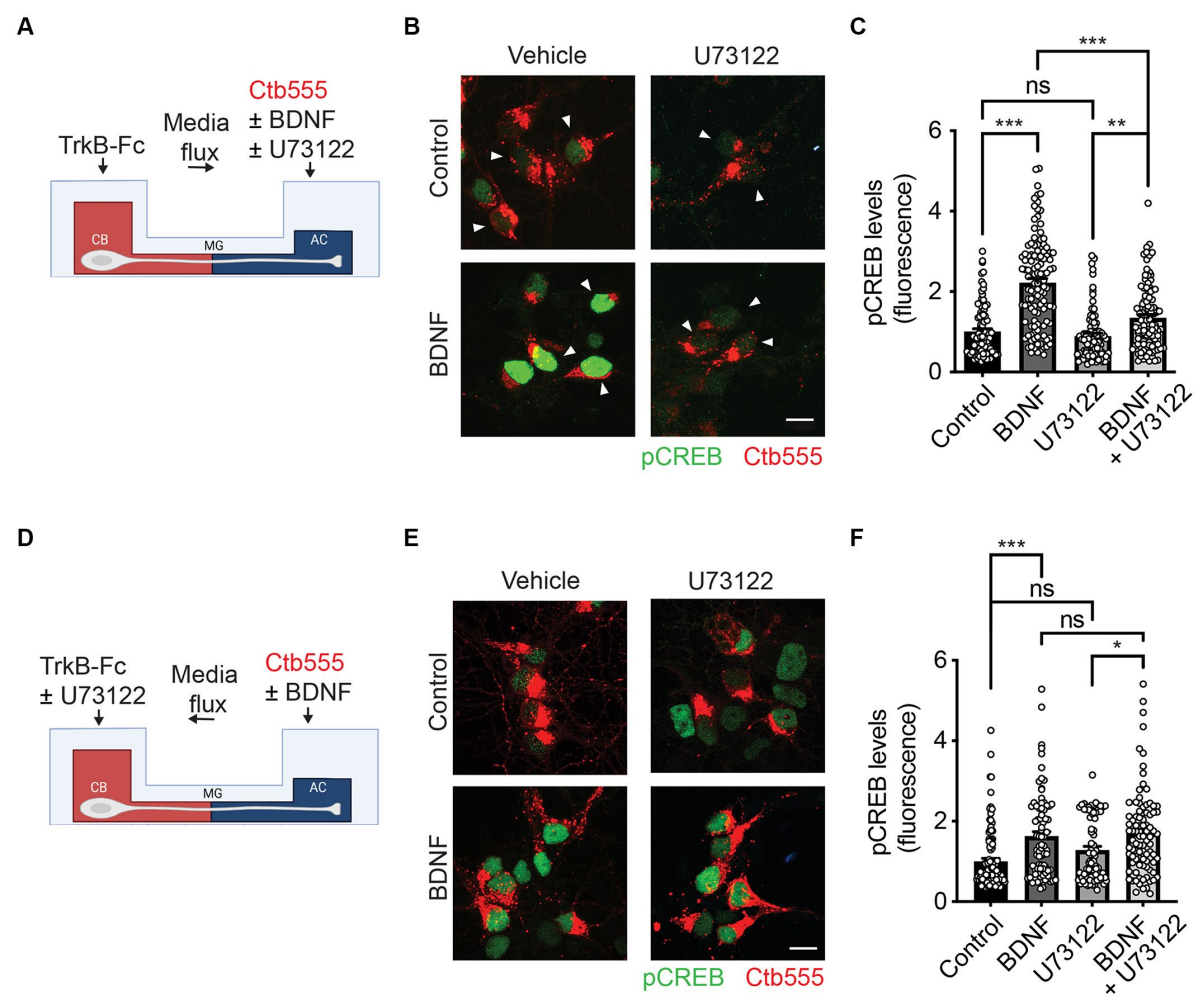
Axonal PLC-γ activity is required for somatodendritic CREB phosphorylation in rat cortical neurons. **(A)** Schematic representation of the protocol used for stimulating neurons. DIV 5 cortical neurons were retrograde labeled with Ctb555 (in red) overnight. At DIV 6, the culture medium was changed to serum-free medium for 90 min in the presence or absence of U73122 (5 μM) in the AC and stimulated with BDNF (50 ng/ml) for 180 min in the AC in the presence or absence of U73122 with the flux toward the AC. Finally, the cultures were fixed, and phosphorylated CREB (pCREB, S133) immunofluorescence was analyzed in cell bodies. **(B)** Representative figures of nuclear pCREB in neurons with or without BDNF stimulation labeled with Ctb555 (in red) added to axons in the presence or absence of axonal U73122 as indicated in **(A)**. Scale bar, 10 μm. **(C)** Quantification of pCREB in the nucleus of neurons labeled with Ctb555 (red) in each condition. *n* = 90–114 neurons from 3 independent experiments as shown in **(B)**. **(D)** Schematic representation of the protocol used for stimulating neurons. DIV 5 cortical neurons were retrograde labeled with Ctb555 (in red) overnight. At 6 DIV, the culture medium was changed to serum-free medium for 90 min in the presence or absence of U73122 (5 μM) in the CB with the flux toward the CB, and then the AC was incubated with BDNF (50 ng/mL) for 180 min. Finally, the cultures were fixed, and pCREB was analyzed in cell bodies. **(E)** Representative figures of nuclear pCREB (in green) in neurons with or without BDNF stimulation labeled with Ctb555 (in red) added to axons in the presence or absence of U73122 in the CB. **(F)** Quantification of pCREB in the nucleus in neurons labeled with Ctb555 in each condition. *n* = 43–60 neurons from 2 independent experiments as indicated in **(D)**. The results are expressed as the means ± SEMs. ns, non significant. *p < 0.05. **p < 0.01, ***p < 0.01. Statistical analysis was performed by one-way ANOVA followed by the Bonferroni correction for multiple comparisons.

TrkB mutations in the PLC-γ docking region impair the phosphorylation of CREB and CaMKIV in non-compartmentalized cultures (Minichiello et al., 2002), suggesting that the activity of PLC-γ is required for CREB phosphorylation. Since we have previously described that axonal stimulation with BDNF promotes nuclear CREB phosphorylation (Bronfman et al., 2014; Moya-Alvarado et al., 2023) we tested whether the activity of PLC-γ was required in the cell body and/or axons for axonal BDNF-dependent CREB phosphorylation. To evaluate CREB phosphorylation, we incubated Ctb555 in the AC overnight to identify all the neurons with crossing axons, in DIV 5 cultures. On the next day, we added BDNF to the AC in the presence or absence of U73122 in the AC or the CB taking care of the media flux direction as indicated in the Figures 2A,D. As we previously reported, axonal signaling of BDNF induced an increase in CREB phosphorylation (Moya-Alvarado et al., 2023). Interestingly, the presence of the PLC-γ inhibitor decreased CREB activation induced by axonal BDNF only when added to the AC (Figures 2B,C), having no effect when added to the CB (Figures 2E,F), which suggests that BDNF/TrkB axonal signaling to cell bodies requires PLC-γ activity mainly for axonal propagation of BDNF signaling.

### PLC-γ is locally activated by BDNF in axons and is required for the transport of BDNF-containing signaling endosomes

**Figure 3:**
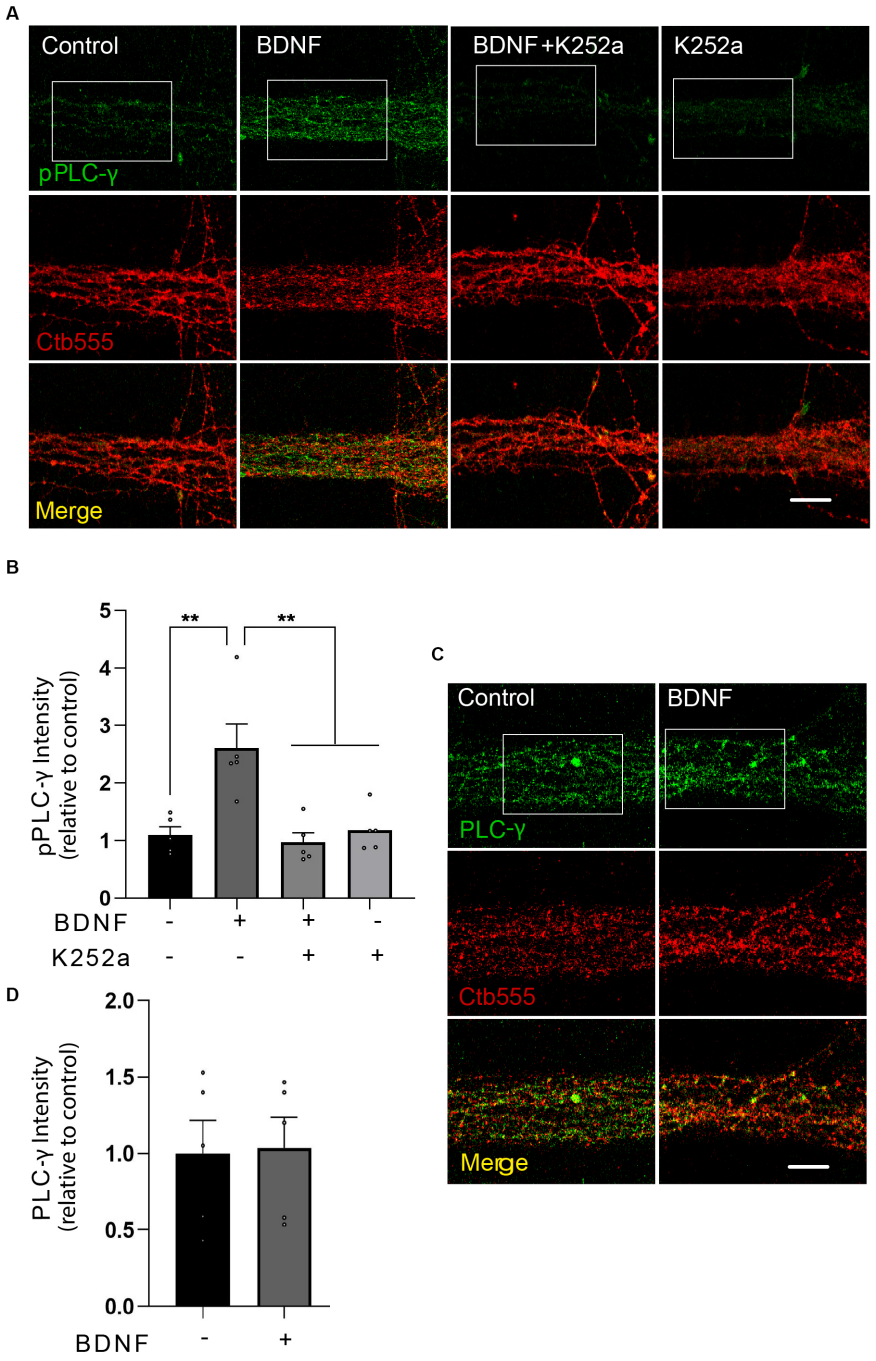
BDNF in axons promotes axonal PLC-γ phosphorylation in rat cortical neurons. **(A)** Representative images of phosphorylated PLC-γ (pPLC-γ, pY783.28, green) in axons of compartmentalized rat cortical neurons left unstimulated (control), stimulated with 50 ng/ml BDNF for 20 min (BDNF) or stimulated with BDNF in the presence of 0.2 μM K252a (BDNF + K252a). Axons were labeled with Ctb555 overnight before treatment to assess correct compartmentalization of the culture. **(B)** Quantification of the immunofluorescence signal associated with pPLC-γ was performed in a rectangular ROI drawn in the border between the beginning of the microgroove and the AC and continuing for 30 μm toward the microgroove that was delimited by Ctb555 fluorescence, shown as a white rectangles in **(A)** and in Supplementary Figure S2A as distal microgroove. *n* = 5 chambers (the value in each chamber corresponds to the average of 5 different microgrooves) performed in five independent cultures. Statistical analysis was performed by one-way ANOVA followed by the Bonferroni correction for multiple comparisons. **p < 0.01. **(C)** Compartmentalized neurons were treated as described in **(A)** (but K252a was not used in this experiment). A representative image of total PLC-γ in the AC quantified as indicated in **(B)** used to assess pPLC-γ immunostaining is shown. **(D)** Quantification of the immunofluorescence signal associated with total PLC-γ. Statistical analysis was performed by Student’s *t*-test. No significant differences were found. Scale bar, 10 μm.

To test whether BDNF activates PLC-γ in axons, we treated distal axons of cortical neurons with BDNF for 20 min and assessed the phosphorylation of PLC-γ by immunofluorescence staining in the microgrooves proximal to the AC. Since phosphorylation of PLC-γ in the tyrosine 728 has been shown to be a reliable proxy for its catalytic activity (Nishibe et al., 1990) we used a phospho-specific antibody to reveal that BDNF increases PLC-γ phosphorylation in the axons of cortical neurons, while immunoreactivity was significantly decreased by treatment with K252a, an inhibitor of the tyrosine kinase activity of Trks (Tapley et al., 1992), confirming the specificity of the staining (Figures 3A,B; Tapley et al., 1992). The total protein levels of PLC-γ, however, remained unaffected by the BDNF treatment (Figures 3C,D and Supplementary Figure S1). Examination of PLC-γ phosphorylation in regions proximal to cell bodies or within cell bodies themselves did not reveal any BDNF-dependent augmentation in staining. This observation strongly suggests localized activation of PLC-γ within axons without significant propagation to the cell body. These findings are presented in Supplementary Figure S2.

**Figure 4:**
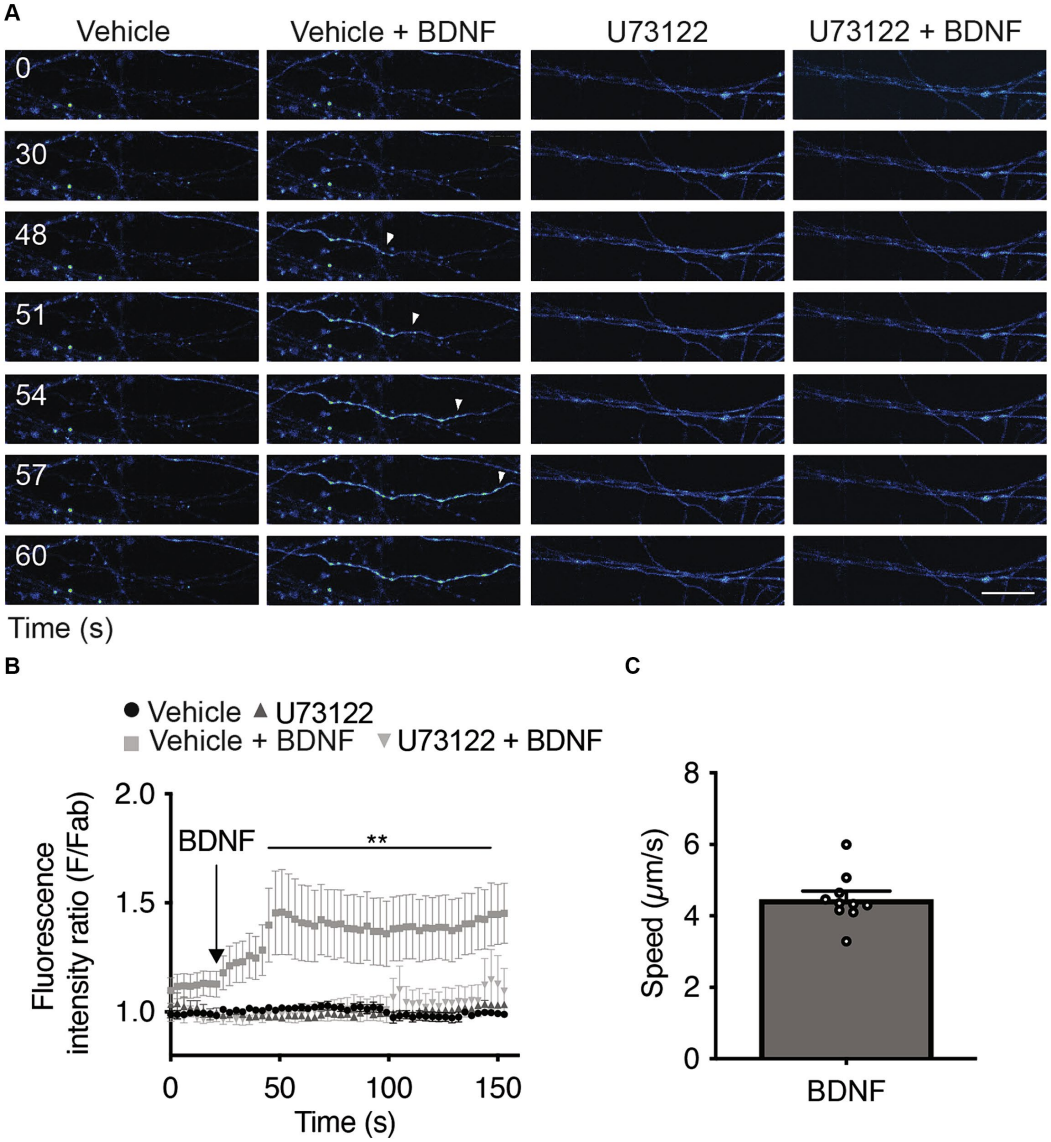
Axonal BDNF promotes an increase in intracellular Ca^2+^ in a PLC-γ-dependent manner in rat cortical neurons. **(A)** Evaluation of Ca^2+^ signaling induced by BDNF. The change in fluorescence intensity associated with Fluo4-AM (2 μM) was used to measure the concentration change in cytosolic Ca^2+^. Representative images of compartmentalized cultures loaded with Fluo4-AM in the AC treated with vehicle or BDNF (50 ng/ml) in the AC with or without U73122 (5 μM) pretreatment. Live-cell imaging of each axonal field in the AC recorded before BDNF treatment (0 s) and during 60 s of BDNF treatment. Scale bar, 10 μm. **(B)** Mean Fluo4-AM fluorescence intensity (±SEM) for each treatment at different snapshot times. Fab represents the average fluorescence of the baseline. We calculate the average of the calcium recordings for the vehicle or U17322 treatments, and subsequently standardize the data of the baseline, BDNF and BDNF + U17622 calcium recordings by these respective values. *n* = 8–12 axons from three independent compartmentalized cultures. Statistical analysis was performed by two-way ANOVA followed by the Bonferroni correction for multiple comparisons. **p < 0.01. **(C)** Quantification of the velocity of Ca^2+^ back-propagation of the fluorescence signal associated with Fluo4-AM under BDNF conditions.

Since the activation of PLC-γ by TrkB leads to an increase in intracellular Ca^2+^ (Zirrgiebel et al., 1995), we assessed whether local stimulation of axons with BDNF increases cytosolic Ca^2+^ in a PLC-γ-dependent manner. We loaded the neurons with Fluo-4 AM, a cell permeant Ca^2+^ indicator (Cheng et al., 2017), and recorded Fluo-4 AM fluorescence in the axons 1 min after BDNF addition to study the immediate effect of BDNF on axonal Ca^2+^ levels. Incubating axons with BDNF resulted in a single-point Ca2+ increase signal that subsequently extended retrogradely, covering the entire axon within the imaging field (Figure 4A). The presence of U73122 completely eliminated the increase in cytosolic Ca^2+^ induced by BDNF stimulation (Figures 4A,B and Supplementary Videos S1–S4). This observation suggests that the observed Ca^2+^ signal relies on PLC-γ activity and is attributed to an increase on intracellular Ca^2+^ from intracellular stores and/or alternatively from the extracellular space. Although intensity and triggering of the local transients were largely heterogeneous, we measured the propagation speed of the retrograde Ca^2+^ signal considering the initial point of Ca^2+^ increase until the last point observable in the video recording; the average measured speed was 4.47 ± 0.15 μm/s (Figure 4C).

**Figure 5:**
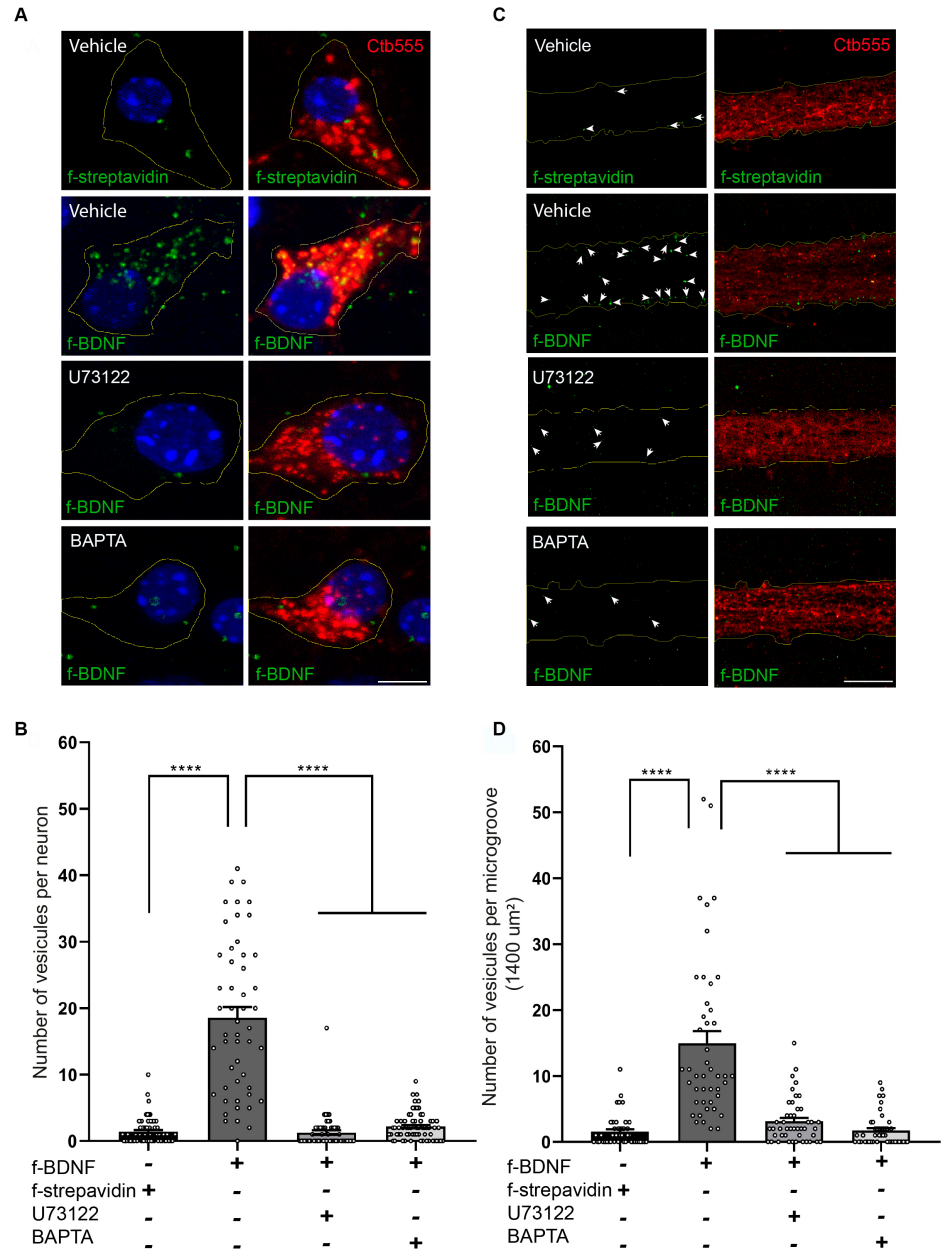
Axonal PLC-γ activity and intracellular Ca^2+^ are required for axonal BDNF-containing signaling endosome generation and retrograde transport to cell bodies in mouse cortical neurons. **(A)** DIV 7 compartmentalized cortical neurons were retrograde labeled with Ctb555 (red) overnight. At DIV 8, the cell body compartment was treated with TrkB-Fc, and the AC was treated with DyLight 488-labeled streptavidin (f-streptavidin, green) alone or with biotinylated BDNF conjugated to DyLight 488-labelled streptavidin (f-BDNF, green), as indicated in the methodology section, for 6 h in the absence or presence of vehicle or 5 μM U73122 (U73122) or 20 μM BAPTA-AM (BAPTA). Then, the cells were fixed, mounted in Mowiol containing Hoechst (blue) and prepared for confocal microscopy. Scale bar, 5 μm **(B)** Quantification of green-labelled vesicles in cell bodies of neurons containing Ctb555. Only vesicles larger than 200 nm^2^ were considered for the analysis. Forty-five neurons from three independent compartmentalized cultures were considered. Statistical analysis was performed by one-way ANOVA followed by the Bonferroni correction for multiple comparisons. ****p < 0.0001. **(C)** Left panels, representative images of f-streptavidin or f-BDNF (green) associated fluorescence. White lines are selecting the region of the microgroove label with Ctb555 (red) shown in the right panels. White arrows indicate green-labelled vesicles. Right panels, Representative images of axons (labeled with Ctb555, red) in microgrooves of neurons treated as described in **(A)**. Scale bar, 5 μm. **(D)** Quantification of green-labelled vesicles in microgrooves of axons containing Ctb555. Forty-five microgrooves from three independent compartmentalized cultures were considered for the analysis. Statistical analysis was performed by one-way ANOVA followed by the Bonferroni correction for multiple comparisons. ****p < 0.0001.

We then evaluated whether this BDNF-induced local PLC-γ signaling impacted retrograde transport of BDNF-containing endosomes by using our previously established assay of fluorescent BDNF retrograde transport in compartmentalized cultures of mouse cortical neurons, where we have shown that BDNF is transported together with active TrkB receptors (Moya-Alvarado et al., 2023). Consistent with the abovementioned results, the transport of BDNF-containing endosomes was evaluated in both the soma and the axonal compartment in the microgrooves. Using U73122 or BAPTA to block PLC-γ or intracellular calcium increase, respectively, we found that transport of BDNF is dependent on both PLC-γ activity and intracellular Ca^2+^ levels (Figures 5A–D). We also quantified the fluorescence associated with Ctb in cell bodies following the various treatments. We observed that the presence of BDNF increased the transport of Ctb, while the presence of U173122 and BAPTA reduced this BDNF-associated increase in Ctb transport, with no effect on basal levels (Supplementary Figures S3, S4). These findings align with previous research indicating that axonal depolarization enhances Ctb transport in a BDNF/TrkB-dependent manner, and that TrkB and Ctb co-localize in axonal vesicles (Wang et al., 2016), as well as our own observations demonstrating colocalization of BDNF and Ctb in axonally transported vesicles (Moya-Alvarado et al., 2024).

### PLC-γ activity and calcium are required for BDNF-dependent TrkB internalization

**Figure 6:**
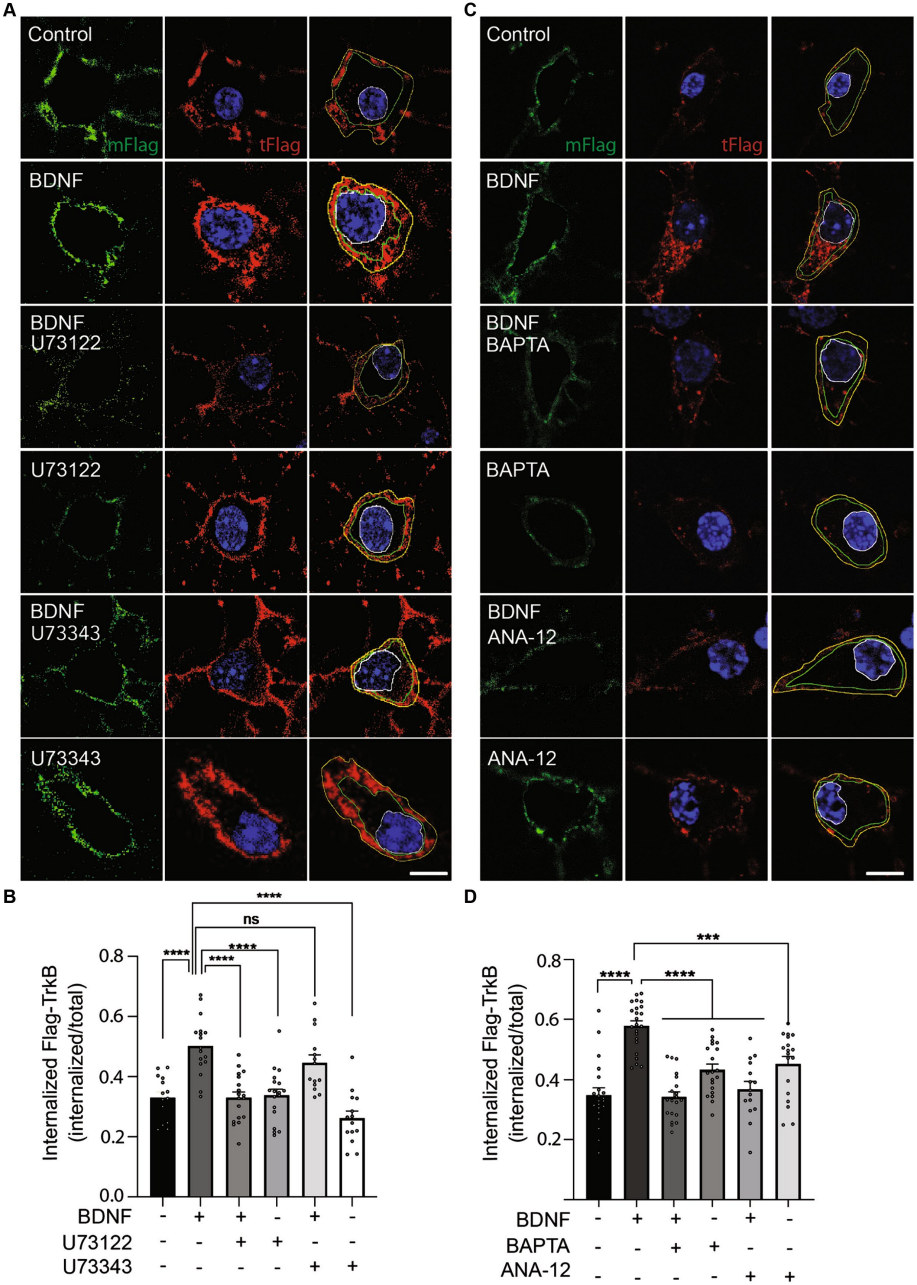
PLC-γ activity and intracellular calcium are required for TrkB internalization in mouse cortical neurons. Neurons (DIV 6) were transfected with a plasmid expressing Flag-TrkB. After 48 h, the neurons were incubated with an anti-Flag antibody. The neurons were treated with BDNF (50 ng/ml) in the presence or absence of U73122 (5 μM) or BAPTA-AM (5 μM) or ANA-12 (10 μM) or BDNF (50 ng/ml) in the presence or absence of U73343 (5 μM) for 20 min to induce endocytosis at 37°C. Finally, the neurons were fixed, and the Flag epitope was detected by immunostaining as indicated in Supplementary Figure S4A. The plasma membrane associated Flag antibody was recognized with an anti-mouse Alexa488 (mFlag, green) before permeabilizing cells, then, cells were permeabilized and the total Flag epitope was recognized with an anti-mouse Alexa555 (tFlag). **(A)** Representative images of the endocytosis of Flag-TrkB in control cells treated with BDNF, U73122, U73343, BDNF with U73122 or BDNF with U73343. The figure shows plasma membrane associated Flag-TrkB (in green), and total Flag-TrkB in red, blue is the nucleus labelled with Hoechst. The yellow line is limiting the outer border of the cell, the green line is limiting membrane versus cytosolic associated Flag-TrkB and the white one the nucleus. Scale bar, 5 μm. **(B)** Quantification of internalized Flag-TrkB was achieved by dividing the fluorescence associated with internalized Flag-TrkB (only red fluorescence between the green and white lines) by the fluorescence intensity associated with total Flag-TrkB. *n* = 14–18 neurons from 3 independent experiments. Scale bar, 5 μm. **(C)** Representative images of the endocytosis of Flag-TrkB in control cells treated with BDNF, BAPTA-AM, ANA-12, BDNF with BAPTA-AM or BDNF with ANA-12. The figure shows plasma membrane associated Flag-TrkB (mFlag, in green), and total Flag-TrkB (tFlag, in red), blue is the nucleus labelled with Hoechst. The green line is limiting membrane versus cytosolic associated Flag-TrkB and the white one the nucleus. Scale bar, 5 μm. **(D)** Quantification of internalized Flag-TrkB in neurons treated with BAPTA-AM and ANA-12. n = 15–23 neurons from 3 independent experiments. The results are expressed as the mean ± SEM. ***p < 0.001; ****p < 0.0001. Statistical analysis was performed by one-way ANOVA followed by Bonferroni’s post-test for multiple comparisons.

Neurotrophin binding to Trks results in the internalization of the receptor–ligand complex into endosomes, where the receptor–ligand complex continues signaling (Beattie et al., 1996; Bronfman et al., 2014). Interestingly, endocytosis of both epidermal growth factor receptor (EGFR) (Delos Santos et al., 2017) and TrkA (Bodmer et al., 2011) is regulated by PLC-γ. Furthermore, TrkB internalization is regulated by neuronal activity and the influx of Ca^2+^, a second messenger downstream of PLC-γ activation (Du et al., 2003). Considering the observation that retrograde transport of BDNF-containing signaling endosomes depends on PLC-γ activity and intracellular Ca^2+^stores (Figure 5), we hypothesized that an effect of PLC-γ activity on TrkB internalization could regulate the assembly of signaling endosomes for retrograde transport. To specifically study whether PLC-γ activity is required for TrkB internalization, we performed an immunoendocytosis assay in non-compartmentalized primary cultures of mouse cortical neurons. Mouse and rat cortical neurons were transfected with a plasmid driving the expression of a TrkB receptor amino terminally tagged with a Flag epitope (Flag-TrkB) as previously described (Lazo et al., 2013; Supplemenatry Figure S4A). Forty-eight hours later, we treated the neurons with an anti-Flag antibody at 4°C. Next, we stimulated mouse cortical neurons with BDNF in the presence or absence of the PLC-γ inhibitor U73122. BDNF increased TrkB internalization by approximately 50% of the basal level, an effect that was significantly reduced when PLC-γ activity was inhibited by U73122 (Figures 6A,B). To control for off target effects of the PLC-γ inhibitor we used the drug U73343, a close structural analog of U73122 that has been extensively used as a negative control for PLC-γ inhibition (Bleasdale et al., 1990; Smith et al., 1990; Smallridge et al., 1992). Neurons treated with BDNF in the presence of the negative control U73343 showed no reduction in receptor internalization as expected (Figures 6A,B). Since U73122 acts as an antagonist of PLC-γ downstream of receptor activation, it is not expected to affect BDNF-dependent phosphorylation of PLC-γ, which we confirmed in western blot analysis of mouse cortical neurons treated with or without BDNF in the presence or absence of U73122 and U73343 (Supplementary Figures S1A,B). Consistently, the kinase inhibitor K252a, which reduces BDNF-dependent TrkB activation, completely abolished BDNF-induced phosphorylation of TrkB (Supplementary Figures S1A,B). Since we have previously shown similar TrkB trafficking capabilities on mouse cortical neurons compared to rat cortical neurons (Moya-Alvarado et al., 2023), we repeated the immunoendocytosis assay in rat cortical neurons. Similar to mouse cortical neurons, rat cortical neurons transfected with Flag-TrkB showed a basal level of recombinant TrkB internalization that was increased by 20 min of BDNF treatment (Supplementary Figures S4B,C), while U73122 almost completely abolished BDNF-dependent internalization of TrkB. In contrast, the inhibitor did not influence the basal levels of Ctb555 internalization (Supplementary Figure S4D).

**Figure 7:**
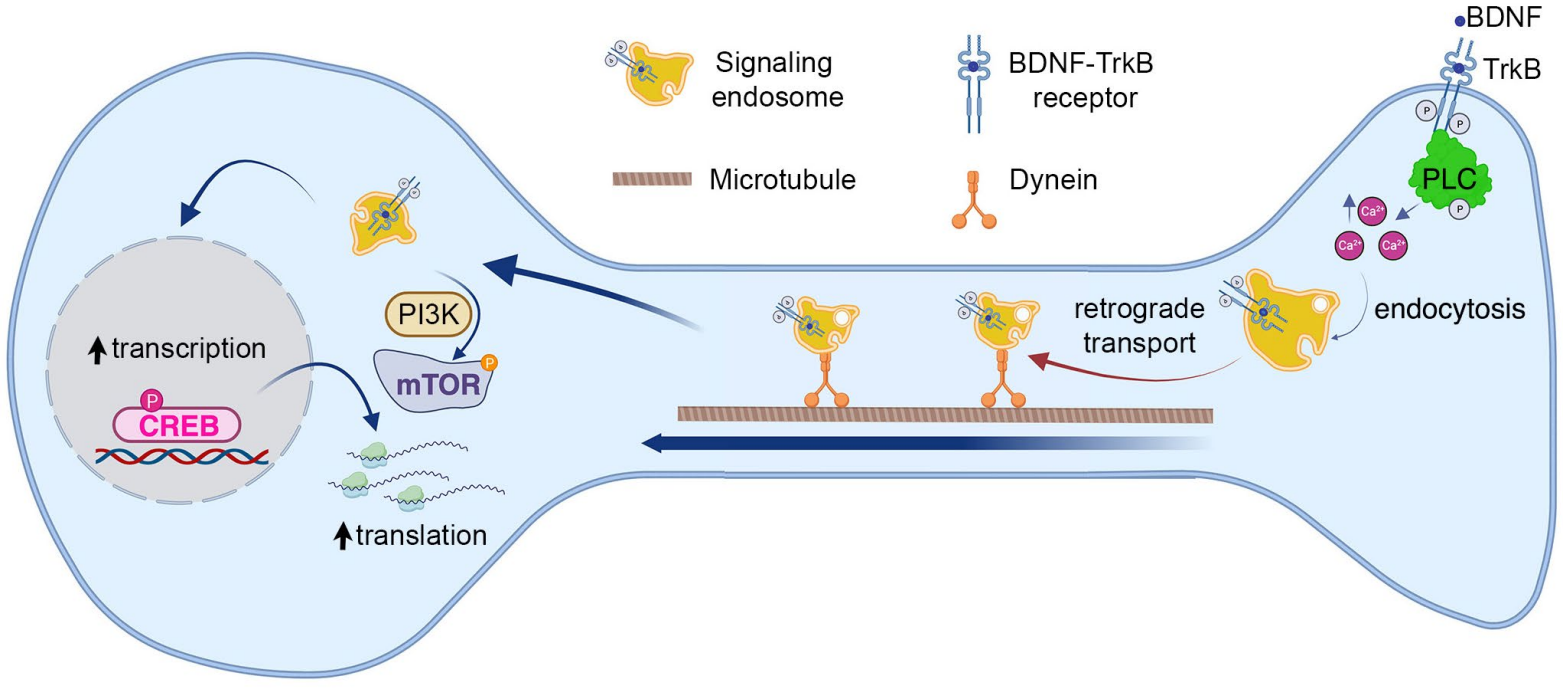
Model summarizing the role of known BDNF–TrkB signaling pathways on long-distance BDNF signaling. The findings from our study suggest that BDNF in the axon activates TrkB receptors and PLC-γ, leading to an increase in intracellular calcium concentration (Ca^2+^). This, in turn, promotes the endocytosis of the receptor and the formation of signaling endosomes. The first step in this process enables the retrograde transport of signaling endosomes to the cell body, contributing to an elevation in CREB phosphorylation and dendritic arborization. Notably, we observed that cell body PLC-γ activity was not essential for CREB phosphorylation induced by axonal BDNF. In previous research, we demonstrated that PI3K activity is not necessary for endocytosis or transport of signaling endosomes but plays a crucial role in mTOR-dependent translation of CREB target genes (Moya-Alvarado et al., 2023). This process was also needed for dendritic arborization induced by BDNF in the axons.

Our work so far suggest a model where BDNF/TrkB signaling activates PLC-γ, which in turn increases intracellular calcium and regulates endocytosis of TrkB receptor. To test this possibility, we evaluated BDNF-induced TrkB-flag internalization in the presence of the cell permeable calcium chelator BAPTA-AM (Collatz et al., 1997) and a specific TrkB kinase inhibitor, ANA-12 (Cazorla et al., 2011). These results showed that endocytosis of TrkB in mouse cortical neurons depends on both availability of intracellular calcium and TrkB kinase activity (Figures 6C,D), supporting a model where BDNF in axons augmented PLC-γ activity, process that in turn increases the levels of intracellular calcium stimulating endocytosis of BDNF/TrkB receptors. In turn, BDNF/TrkB internalization favors the transport of BDNF signaling endosomes and nuclear signaling (Figure 7).

## Discussion

The spatial regulation of various BDNF signaling pathways in distinct subcellular neuronal domains has received limited exploration. In prior studies conducted by our group, we observed that BDNF-mediated long-distance signaling from axons enhances cellular responses, resulting in dendritic arborization. This process necessitated PI3K activity in cell bodies but not in axons, where signaling endosomes were transported independently of PI3K activity (Figure 7) (Moya-Alvarado et al., 2023). Here, we show that signaling endosomes to form required BDNF-induced PLC-γ activity and intracellular Ca^2+^ in axons since both activities were necessary for ligand-dependent endocytosis of TrkB (Figure 7). Consistently, inhibition of PLC-γ activity or reduction of intracellular Ca^2+^ after BDNF addition to axons reduced BDNF-endosomal accumulation in cell bodies, CREB activation in the nucleus, and dendritic arborization. In our studies, the timing of each experiment has been designed to maximize sensitivity rather than describe the cellular cascade. Therefore, we used different time points to measured BDNF accumulation in cell bodies (6 h), CREB phosphorylation in the nuclei (3 h) and dendritic arborization (48 h) after BDNF treatment in axons.

In this study we have favored pharmacological inhibitors of PLC-γ-Ca^2+^ BDNF downstream signaling pathway over genetic tools. This approach allowed us to inhibit enzymatic or biological activities in a localized manner, that is, in the cell bodies or the axonal compartment of microfluidic cultures, which is a type of experimental design that current genetic tools do not allow. Undoubtedly, pharmacological inhibitors have an explicit limitation of selectivity, but they constitute crucial tools to identify molecular targets and mechanisms. Future development of a more selective toolkit to assess local activities in neuronal cells is warranted; such as optogenetic inhibition, as it has been used for RabGTPases and the TrkA receptor (Nguyen et al., 2016; Khamo et al., 2019; Cornejo et al., 2020).

Previous studies in retinal ganglionic cells, of the *Xenopus laevis* optic tectum showed that stimulation with BDNF promotes a potentiation of retinotectal synapses in a retrograde-dependent manner. This process was dependent on the activity of TrkB and PLC-γ, suggesting that there are synaptic modifications that may be causally linked to structural modification of RGC dendrites after hours of BDNF stimulation in the axons (Lom et al., 2002; Du and Poo, 2004). Therefore, it is possible to speculate that PLC-γ activity is required for endocytosis of TrkB receptors in axons of other circuits, such as in axons of retinal ganglionic cells.

In the same context, we observed that axonal but not cell body activity of PLC-γ is required for dendritic arborization and CREB phosphorylation induced by BDNF axonal signaling in cortical neurons, suggesting that PLC-γ has an axonal local role in cortical axons. Our data suggest that this regulatory role involves endocytosis of TrkB receptors leading to signaling endosome formation in distal axons (Figure 7). Inhibition of PLC-γ in cell bodies did not reduce CREB activation induced by the addition of axonal BDNF, indicating that PLC-γ is required for a step during the initiation and propagation of axonal signaling endosomes, but does not participate in CREB phosphorylation when BDNF signaling has reached the cell body. It has been shown that mutation of the TrkB docking site for PLC-γ downregulates BDNF-induced activation of CREB in non-compartmentalized cortical neuronal cultures after 15 min of BDNF exposure (Minichiello et al., 2002); however, this study did not address whether sustained activation of CREB was impaired. Therefore, it is possible that BDNF signaling propagation from different locations, including the somatodendritic and the axonal domain and different signaling pathways globally, contributes to sustained CREB activation, allowing CREB-dependent dendritic arborization (Moya-Alvarado et al., 2023).

PLC-γ has been described as a regulator of TrkA and EGFR endocytosis, and two different mechanisms have been reported for this PLC-γ-dependent internalization. For TrkA, Ca^2+^ contributes to enhanced clathrin-mediated endocytosis in neurons due to dephosphorylation of dynamin 1 by calcineurin a Ca^2+^-dependent protein phosphatase (Bodmer et al., 2011). On the other hand, calcineurin and dynamin 1 are dispensable for the internalization of EGFR. In this case, Ca^2+^ ions recruit synaptojanin 1 (a lipid phosphatase) to clathrin-coated pits in a PKC-dependent manner induced by the activation of PLC-γ (Delos Santos et al., 2017). The mechanism by which PLC-γ/Ca^2+^ regulates TrkB internalization in cortical axons will be a matter for further studies. While regulation of TrkB endocytosis is expected to have a crucial impact on the formation of signaling endosomes, it is interesting to notice that both calcineurin and synaptojanin 1 have a role in endosomal transport as well (Fasano et al., 2018; Scaramuzzino et al., 2022) keeping open the possibility of an integration between mechanisms regulating endocytosis, sorting and axonal transport of signaling carriers (see below) (Figure 7).

In non-compartmentalized cultures, bath application of BDNF promotes a strong increase in the frequency of global Ca^2+^ transients (He et al., 2005; Lang et al., 2007), but local BDNF application induces a fast and spatially restricted signal in dendrites (Lang et al., 2007). We observed that BDNF axonal stimulation led to an increase in intracellular Ca^2+^. Similar to the observations in dendrites, we initially noted a localized point of Ca^2+^ elevation, followed by an extended retrograde transient signal across the axons in the recording zone. Notably, despite TrkB being located on the surface of axons, the generation of Ca^2+^ waves occurred at heterogeneous locations along the axons. One intriguing hypothesis, which warrants further experimental validation, is that these locations correspond to sites where TrkB receptors are internalized and therefore concentrated. Internalized TrkB would locally increases IP3 levels, potentially activating intracellular IP3 receptors and inducing calcium release from intracellular axonal stores. However, it is important to emphasize that these interpretations remain speculative. Of note, we observed that the velocity of the Ca^2+^ waves was faster than the average velocity of fluorescent BDNF (1.11 ± 0.05 μm/s) (Xie et al., 2012) and (1.5 ± 0.3 μm/s) for GFP-TrkB (Goto-Silva et al., 2019), suggesting that this Ca^2+^ increase is independent of the retrograde transport of the signaling endosome. Interestingly, it was recently shown that calcineurin is in the cytosolic part of signaling endosomes, where it dephosphorylates huntingtin in a Ca^2+^-dependent manner to favor the retrograde transport of TrkB endosomes. This suggests that the Ca^2+^ waves induced by BDNF contribute to the retrograde trafficking of axonal TrkB endosomes (Scaramuzzino et al., 2022). Other possibility for Ca^2+^ waves to contribute to signaling endosome transport is by increasing JIP3-dependent dynactin/dynein recruitment to signaling endosomes (Cavalli et al., 2005; Rishal and Fainzilber, 2014). Further experiments studying BDNF-induced calcium waves and signaling endosomes transports may help elucidating this phenomenon.

In summary, this study reveals a specific function for PLC-γ signaling and axonal calcium transients in cortical neurons, regulating the propagation of BDNF/TrkB signaling from distal axons to the cell body to increase CREB-dependent dendritic branching. While the direct contribution of calcium signaling to post-endocytic sorting and axonal transport of TrkB remain to be clarified in future studies, we showed that BDNF-induced endocytosis of TrkB receptors is regulated by PLC-γ activity and availability of intracellular Ca^2+^ (Figure 7), offering a working mechanism by which TrkB- PLC-γ signaling modulates the formation of retrograde signaling carriers.

## Methodology

### Primary culture of cortical neurons

Embryonic cortical neurons (16–18 days of gestation) were obtained from mice (C57Bl/6 J) and rats (*Rattus norvegicus*) housed in the animal facilities of our institutions. Pregnant animals were euthanized under deep anesthesia according to bioethical protocols approved by the Bioethics Committee of the Pontificia Universidad Catolica (protocol ID:180822013) de Chile and Universidad Andres Bello (act of approval 022/2019 and 009/2022).

Rat and mouse cortical tissues were harvested and dissociated into single cells in Hank’s balanced salt solution (HBSS; Thermo-Fisher, cat# 14025134). After disaggregation, the neurons were resuspended in modified Eagle’s medium (MEM) supplemented with 10% horse serum (HS) (MEM/HS, Thermo-Fisher, cat# 16050122) and seeded in microfluidic chambers at a low density (40–50 × 10^3^ cells/chamber) or in mass culture at a density of 35 × 10^3^ cells/well on 12 mm coverslips or 1.5 × 10^6^ cells/60 mm plate. After 4 h, the culture medium was replaced with neurobasal medium (Thermo-Fisher, cat# 21103049) supplemented with 2% B27 (Life Technologies, cat# 17504044), 1x GlutaMAX (Thermo-Fisher, cat# 35–050-061) and 1x penicillin/streptomycin (Thermo-Fisher, cat# 15140–122). The proliferation of nonneuronal cells was limited using cytosine arabinoside (0.25 μg/ml AraC, Sigma–Aldrich, cat# C1768) when MEM/HS was replaced with neurobasal medium (Shimada et al., 1998; Taylor et al., 2003; Moya-Alvarado et al., 2023). Figures 1–4 and Supplementary Figures S2, S4 were performed in rat cortical neurons and Figures 5, 6 and Supplementary Figures S1, S3 were performed in mouse cortical neurons.

### Microfluidic devices

We thank the lab of Professor Eran Perlson at the Department of Physiology and Pharmacology of the Faculty of Medicine of Tel Aviv University for providing the molds to prepare the compartmentalized chambers (Gluska et al., 2016). The microfluidic chambers were prepared with SYLDGARD™ 184 silicone elastomer base (Poirot, cat# 4019862) according to the manufacturer’s instructions. Two days before primary cultures plating, glass coverslips (25 mm) were incubated with poly-D-lysine (0.1 mg/ml, Corning, cat# 354210). The next day, poly-D-lysine was washed, and a microfluidic chamber with a 400 μm microgroove was placed on the coverslip. Then, laminin (2 μg/ml in water, Invitrogen, cat# 23017015) was added to the chamber. The same day, the primary culture laminin solution was changed to DMEM/HS medium (Dulbecco minimum essential medium supplemented with 10% horse serum, 1x GlutaMAX and 1x antibiotic/antimycotic, Thermo-Fisher, cat# 15240062).

### Quantification of BDNF-induced dendritic arborization in rat cortical neurons

Cortical neurons (DIV 6) were transfected with 0.5 μg of the plasmid containing EGFP (CA, USA) using 0.8 μl of Lipofectamine 2000 (Invitrogen, cat# 11668–019) in 30 μl of Opti-MEM (Thermo-Fisher, cat# 11058021). After 2 h, the Opti-MEM medium was replaced with neurobasal medium, and the cells were incubated for 1 h. The CB was incubated with neurobasal medium supplemented with TrkB-Fc (100 ng/mL, B&D system, 688TK) for all treatments. The drugs were incubated in the CB or AC. In the AC, U73122 (5 μM, Sigma–Aldrich cat# U6756) was used. After 1 h, BDNF (50 ng/ml, Alomone, cat# B-250) was added to the AC together with fluorescent subunit B of cholera toxin (Ctb, 1 μg/mL, Thermo-Fisher, cat# C34777). After 48 h, neurons were washed with PBS 37°C and then fixed with fresh 4% PFA-PBS at 37°C for 15 min [paraformaldehyde (Sigma–Aldrich, cat# 158127) in PBS]. Then, the chamber was removed, and the neurons were permeabilized and blocked with 5% BSA (Jackson, cat# 001–000-162) and 0.5% Triton X-100 (Sigma–Aldrich, cat# 234729) in PBS and then incubated with anti-MAP2 (1:500, Merck-Millipore, cat# AB5622) in incubation solution (3% BSA with 0.1% Triton X-100 in PBS). After washes were performed (3x buffer), the neurons were treated with a donkey anti-mouse Alexa 647 antibody (1:500, Molecular Probes, cat# A-31571) in incubation solution and mounted for fluorescence microscopy visualization using Mowiol 4–88 (Calbiochem, cat# 475904).

Dendritic arborization was analyzed in cortical neurons labeled with Ctb and labeled for EGFP and MAP2. Primary dendrites, branching points and Sholl’s analysis (Sholl, 1953) were quantified (see below). For visualization, confocal microscopy using a Nikon Eclipse C2 confocal microscope equipped with a digital camera connected to a computer with Software NIS-Elements C was used. Five to seven optical sections (0.5 μm thick) from the whole cells, using the EGFP associated fluorescence, were acquired using a 60x objective at a resolution of 1024 × 1024 pixels along the *z*-axis. The *z*-stacks were integrated, and the images were segmented to obtain binary images. Ten concentric circles with increasing diameters (5 μm each step) were traced around the cell body, and the number of intersections between dendrites and circles was counted and plotted for each diameter. Analysis was performed using the ImageJ plugin developed by the Anirvan Gosh Laboratory^1^. The total primary dendrites and branching points of all dendrites were manually counted from the segmented images.

### Evaluation of CREB protein phosphorylation by immunofluorescence staining in rat cortical neurons

To evaluate the levels of phosphorylated CREB (pCREB) in the nucleus, experiments were performed as indicated in Moya-Alvarado et al. (2023). In brief, rat cortical neurons (DIV 6) were incubated with Ctb555 (Ctb, 1 μg/ml) overnight. Next, cortical neurons (DIV 7) were incubated with neurobasal medium containing TrkB-Fc (100 ng/ml) in the cell body compartment. To inhibit PLC-γ, we used the drug U73122 (5 μM) in the AC or cell body compartment depending on the experimental design. After 1 h of inhibitor pretreatment, BDNF (50 ng/mL) was added to the AC. After 180 min, samples were fixed with 4% PFA supplemented with a phosphatase inhibitor (1x) for 15 min. Permeabilization and blocking were performed with bovine serum albumin (BSA, 5%), Triton X-100 (0.5%) for 1 h in PBS. The samples were incubated with antibodies overnight at 4°C in the presence of BSA (3%) and Triton X-100 (0.1%) in PBS. An anti-phospho-CREB antibody (1:500, Cell Signaling cat# 9198) was used. The samples were incubated with secondary antibodies for 90 min in PBS containing BSA (3%) and Triton X-100 (0.1%). Finally, the samples were incubated with Hoechst 33342 (5 μg/mL, Invitrogen, cat# 62249) and mounted in Mowiol 4–88. Neurons were visualized by confocal microscopy using a Nikon Eclipse C2 confocal microscope equipped with a digital camera connected to a computer with NIS-Elements C software. Five to seven optical sections (0.5 μm thick) from the whole cells of Ctb555-positive neurons were acquired using a 60x objective at 1024 × 1024 pixel resolution along the *z*-axis.

### Evaluation of BDNF-induced phosphorylation of PLC-γ in axons of rat cortical neurons

To measure the levels of phosphorylated PLC-γ (pPLC-γ) in axons after BDNF treatment, rat cortical neurons (DIV 6) in compartmentalized cultures were incubated with Ctb555 (1 μg/mL) overnight. The next day (DIV 7), the neurons were incubated with neurobasal medium containing TrkB-Fc (100 ng/ml) in the cell body compartment, and the AC was pre-incubated for 1 h with vehicle or 0.2 μM K252a (Tocris cat# 1683) (Tapley et al., 1992). Then, BDNF (50 ng/ml) was added to the AC in the presence or absence of K252a. After 20 min, the samples were fixed with 4% PFA/4% sucrose supplemented with a phosphatase inhibitor (Pierce, cat# A32957) for 15 min at room temperature (RT). Permeabilization and blocking were performed with PBS containing BSA (5%) and Triton X-100 (0.5%) for 1 h. The samples were incubated with antibodies overnight at 4°C in PBS containing BSA (3%) and Triton X-100 (0.1%). Anti-phospho-PLC-γ (pY783.28, 1:200, Santa Cruz cat# sc-136186) and anti-PLC-γ1 (PLC-γ1, 1:200, Santa Cruz, cat# sc-7290) antibodies were used. The samples were incubated with secondary antibodies for 90 min in PBS containing BSA (3%) and Triton X-100 (0.1%). Finally, the samples were mounted in Mowiol 4–88 supplemented with Hoechst 33342 (5 μg/mL, Invitrogen, cat# 62249). To measure the level of total PLC-γ, K252a was not used.

Axons were visualized by spinning disk microscopy (Olympus DSU Spinning Disc) on a system equipped with a motorized stage and a digital camera connected to a computer running Olympus cellSens dimension software V 1.14. Whole axons positive for Ctb555 were visualized using an oil immersion (60x) objective at a resolution of 1024×1024 pixels along the z-axis. For quantification, the region of interest (ROI) was defined as the microgroove region nearest the AC. Five images were acquired per chamber (5 microgrooves were evaluated per chamber). For quantification, Image J 1.53 t software was used. From each sample, the image with the highest Ctb55 signal along the *z*-axis was selected. The image was separated in two channels (red, Ctb555 and green, pPLC-γ or total PLC-γ) and converted to 8 bits for analysis. Then, the ROI was defined as a rectangle 15 μm high and 30 μm long, and the mean fluorescence intensity was calculated (total fluorescence intensity divided by 450).

### Measurement of axonal intracellular Ca^2+^

The changes in intracellular Ca^2+^ concentrations were examined by Fluo4-AM staining. Neurons were incubated with Fluo4-AM (2 μM, Invitrogen cat# F14201) in neurobasal medium for 30 min at 37°C and then incubated for another 15 min after rinsing with HBSS. Ca^2+^ imaging was performed by confocal microscopy using a Nikon Eclipse C2 confocal microscope equipped with a digital camera connected to a computer with NIS-Elements C software. The chambers were attached to the stage of the microscope and the neurons were excited with a laser at 488 nm, and the intensity of the fluorescence was collected at 525 nm as the Fluo4-AM signal. Neurons were maintained for 5 min and the images were collected for 30 s (0.25 frames/s) in the axonal compartment. Then BDNF (final concentration 50 ng/mL) was added to the axon compartment by pipetting, to then image the same position of the chamber for other 60 s (0.25 frames/s). The fluorescence intensity was analyzed with ImageJ. To measure Ca^2+^ in the axonal terminals in the presence of U73122 (5 μM), neurons were preincubated in the axonal terminal with U73122 after Fluo4-AM loading.

### Quantification of BDNF-containing signaling endosomes in compartmentalized cultures of mouse cortical neurons

To prepare fluorescent BDNF (f-BDNF), commercially available biotinylated BDNF (Alomone Labs, cat# B-250-B) was coupled to DyLight 488-conjugated streptavidin (Invitrogen, cat# 21832) by incubation at 37°C for 20 min; the reaction consisted of 6 μl of a 6 μM solution of DyLight 488-conjugated streptavidin and 2 μl of a 6 μM solution of biotinylated BDNF diluted in 72 μl of neurobasal medium supplemented with 0.1% BSA (molar range 1:1). Then, this solution was diluted in the same buffer to a final concentration of 130 ng/ml BDNF as described in Moya-Alvarado et al. (2023).

Experiments with compartmentalized mouse cortical neurons were performed as described in Moya-Alvarado et al. (2023). In brief, mouse cortical neurons (DIV 7) were incubated with Ctb555 (1 μg/ml) overnight. Next, neurons (DIV 8) were incubated with neurobasal medium containing TrkB-Fc (100 ng/ml) in the CB compartment and with vehicle (DMSO), U73122 (5 μM) or 20 μM BAPTA-AM (Invitrogen, cat# B6769) for 1 h at 37°C in the AC. Then, f-BDNF was added to the AC for 6 h in the presence or absence of the inhibitors. Then, the drugs were removed, and the neurons were fixed in 4% PFA/4% sucrose in PBS for 18 min, washed in PBS and distilled water and mounted in Mowiol 4–88 supplemented with Hoechst 33342 (5 μg/ml). Cell bodies and axons in the microgrooves were visualized using a confocal microscope (Leica SP8). Fifteen to twenty optical sections (0.5 μm thick) from the whole cell body or axons positive for Ctb555 were acquired using an oil immersion (63x) objective at a resolution of 1024 × 1024 pixels along the *z*-axis. For cell bodies and axons, a 5x or 2.5x digital zoom setting was used, respectively. Fifteen images, containing 1–4 cells or at least one microgroove, were acquired per condition in each experiment. For quantification, Image J 1.53 t was used. To create one image, the maximum projection in each channel was generated using the z-stack images, and the background was reduced in the green channel (f-BDNF). The same procedure was performed for all conditions. All f-BDNF (green)-positive vesicles in each cell were selected, and the diameters were calculated. Only vesicles with an area greater than 20*10^−2^ μm^2^ were used for quantification, and the results are expressed as the number of BDNF-positive vesicles per cell or microgroove.

### Flag-TrkB immunoendocytosis assay to evaluate the effect of PLC-γ activity on the internalization of TrkB

Rat or mouse cortical neurons (DIV 6) were transfected with 0.5 μg of the Flag-TrkB plasmid (a gift from Prof. Francis Lee, Weill Cornel Medicine, New York, NY, US) using 1 μl of Lipofectamine 2000 in 100 μl of Opti-MEM per cover. After 48 h, cortical neurons (DIV 8) were pretreated for 1 h with TrkB-Fc (100 ng/ml) at 37°C and were then incubated on ice for 10 min and treated with an anti-Flag antibody (1:500, Sigma–Aldrich, cat# F3040) for 45 min on ice in the presence or absence of 5 μM U73122, 5 μM U74333, 20 μM BAPTA-AM or 10 μM ANA-12 (Sigma-Aldrich, cat# SML0209-5MG) (U74333, BAPTA-AM and ANA-12 were used only in the experiments with mouse cortical neurons). Then, the cortical neurons were washed briefly with RT neurobasal medium and incubated with BDNF (50 ng/ml) in the presence or absence of U73122 for 20 min. The neurons were washed twice with RT PBS and fixed in 4% PFA/4% sucrose in PBS for 15 min at RT. Then, the cells were washed twice with PBS and incubated with a donkey anti-mouse Alexa 488 antibody (1:300, Life Technologies, cat# A-2120) without permeabilization for 1 h. Then, the samples were permeabilized and blocked with a solution of PBS supplemented with 5% BSA and 0.5% Triton X-100 for 1 h at room temperature. After 1 PBS wash, the samples were incubated with a donkey anti-mouse Alexa 555 antibody (1:300, Invitrogen, cat# A-31570) diluted in PBS supplemented with 1% BSA and 0.1% Triton X-100 overnight at 4°C. Finally, the samples were washed in PBS and distilled water and mounted in Mowiol 4–88 supplemented with Hoechst 33342 (5 μg/ml).

Neurons were visualized using a confocal microscope (Leica SP8). Twelve to fifteen optical sections (0.5 μm thick) from whole neurons were acquired using an oil immersion (63x) objective at a resolution of 1024 × 1024 pixels along the z-axis. For quantification, the Image J 1.53t software was used. To quantify the internalization of Flag-TrkB receptors, the optical section passing through the middle of the soma having the most intense red signal was evaluated (anti-mouse Alexa 555). The image was separated in two channels (red, anti-mouse Alexa 555 and green, anti-mouse Alexa 488) and converted to 8 bits for analysis. To control for the red signal associated to non-internalized receptors, the green signal corresponding to the anti-mouse Alexa 488 antibody (Flag epitope present in the surface) was used to define the plasma membrane. Internalized Flag-TrkB was indicated by the red signal associated with the anti-mouse Alexa 555 antibody within the region of the cell excluding the plasma membrane. The total Flag-TrkB content was represented by the total signal associated with the anti-mouse Alexa 555 antibody. To normalize the level of Flag-TrkB expression, the intensity of anti-mouse Alexa 555 fluorescence associated with internalized TrkB was divided by the total fluorescence intensity associated with the anti-mouse Alexa 555 antibody (see Figures 6, 7).

### Western blot analysis of phosphorylated and total PLC-γ

One million five hundred mouse cortical neurons were seeded in 60 mm plates. After DIV 8, the neurons were treated for 1 h with TrkB-Fc (100 ng/ml) in the presence or absence of the inhibitor U73122 (5 μM) or U74333 (5 μM). Then, the plates were treated with BDNF (50 ng/ml) in the presence or absence of the inhibitors. After treatment, the cells were placed on ice and washed in cold PBS twice, and lysates were prepared with RIPA buffer (150 mM sodium chloride, 1.0% Triton X-100, 0.5% sodium deoxycholate, 0.1% sodium dodecyl sulfate, 50 mM Tris (pH 8.0)). The homogenates were placed in an Eppendorf tube and centrifuged for 20 min at 12000 rpm and 4°C in a benchtop centrifuge. Then, the supernatant was placed in an Eppendorf tube on ice, and protein concentrations were quantified. To detect pPLC-γ and total PLC-γ, 20 μg of protein from each sample was loaded into an SDS-PAGE gel and transferred onto PVDF membranes. Membranes were activated with methanol and blocked with 5% BSA (Jackson ImmunoResearch, cat# 001–000-162) in TBST [150 mM NaCl, 20 mM Tris–HCl (pH 7.4), 0.1% Tween® 20] for 1 h. Incubation with the mouse anti- pPLC-γ (1:1000) primary antibody was performed in TBST overnight at 4°C. After three washes with TBST, the membranes were treated with a 1:10000 dilution of a mouse anti-HRP antibody (BioRad, cat# 1706516) for 2 h and developed with a bioluminescence kit (Biological Industries, cat# 20–500-1000B) using an imaging system (GE Healthcare Life Science, ImageQuant™ LAS 500). Then, the membranes were stripped (Restore™ PLUS Western Blot Stripping Buffer, Thermo Scientific, cat# 4630) for 15 min at RT and blocked with 5% nonfat milk (Rockland, cat# b501-0500) in TBST for 1 h. Then, the membrane was incubated with a mouse anti-PLC-γ antibody (1:500, Santa Cruz, cat# sc-7290) overnight. After three washes with TBST, the membrane was treated with a 1:10000 dilution of the mouse-anti-HRP antibody and developed as indicated above. To quantify the bioluminescence associated with each band, the “Gels” plugin in ImageJ 1.53t was used.

### Statistical analysis

The results are expressed as the averages ± standard errors of the mean (SEMs). Sholl’s analysis curves were compared with two-way repeated-measures ANOVA followed by the Bonferroni correction for multiple comparisons. Student’s *t-*test or one-way ANOVA followed by the appropriate multiple comparisons test was performed depending on the number of groups used in each experiment. The details about the specific test used, level of significance and number of replicates are indicated in each figure legend. Statistical analyses were performed using GraphPad Prism 7 (Scientific Software).

## Data availability statement

The original contributions presented in the study are included in the article/Supplementary material, further inquiries can be directed to the corresponding author.

## Ethics statement

Bioethical protocols were approved by the Pontificia Universidad Catolica Bioethics Committee (protocol ID: 180822013) and Universidad Andres Bello (act of approval 022/2019 and 009/2022). The study was conducted in accordance with the local legislation and institutional requirements.

## Author contributions

GM-A designed and performed experiments and drafted the first manuscript. XV-P designed and performed experiments and contributed to draft the manuscript. AA-S designed and performed experiments and contributed to draft the manuscript. OML contributed to review and editing, formal analysis, visualization and conceptualization. FJB contributed to review and editing, data curation, conceptualization and validation. FCB supervised the research, data curation, visualization and conceptualization, drafted the manuscript and funding acquisition. All authors contributed to the article and approved the submitted version.

## Footnotes

^1^ http://biology.ucsd.edu/labs/ghosh/software
