# Supplementary material for: PLC-γ-Ca2+ pathway regulates axonal TrkB endocytosis and is required for long-distance propagation of BDNF signaling
Source: Front Mol Neurosci. 2024 Apr 10;17:1009404. doi: 10.3389/fnmol.2024.1009404 (PMC11040097; doi:10.3389/fnmol.2024.1009404)
Supplement: Supplementary file 5 [file Presentation_1.pdf]

Supplementary Material

PLC-γ-Ca^{+2} pathway regulates axonal TrkB endocytosis and is required for long-distance propagation of BDNF signaling.

Moya-Alvarado, G[3,#&], Valero-Peña X[1&], Aguirre-Soto A[1], Bustos FJ[2], Lazo OM[4], and Bronfman FC[1*].

[1]NeuroSignaling Laboratory, Institute of Biomedical Sciences (ICB), Faculty of Medicine, and Faculty of Life Sciences, Universidad Andres Bello, Echaurren 183, Santiago, 8370146, Chile.

[2]Constantin-Paton Research Laboratory, Institute of Biomedical Sciences (ICB), Faculty of Medicine, and Faculty of Life Sciences, Universidad Andres Bello, Echaurren 183, Santiago, 8370146, Chile.

[3]Faculty of Biological Sciences, Pontificia Universidad Catolica de Chile (UC). Av. Libertador Bernardo O´Higgins 340, Santiago, 8970117, Chile.

[4]Department of Neuromuscular Diseases, UCL Queen Square Institute of Neurology, University College London, United Kingdom.

[#]Current address: Department of Biology, Johns Hopkins University, Baltimore, MD, 21218, USA.

[&]Equal contributors.

*** Correspondence:** francisca.bronfman@unab.cl. Tel: +56 226618358

1 Supplementary Data

Supplementary data 1. Video1. Video supporting Figure 4 panel A, basal axonal calcium imaging in the presence of vehicle (control).

Supplementary data 2. Video2. Video supporting Figure 4 panel A, calcium imaging after adding BDNF.

Supplementary data 3. Video3. Video supporting Figure 4 panel A, basal calcium imaging in the presence of U73122.

Supplementary data 4. Video4. Video supporting Figure 4 panel A, calcium imaging after adding BDNF in the presence of U73122.

1.1 Supplementary Figures

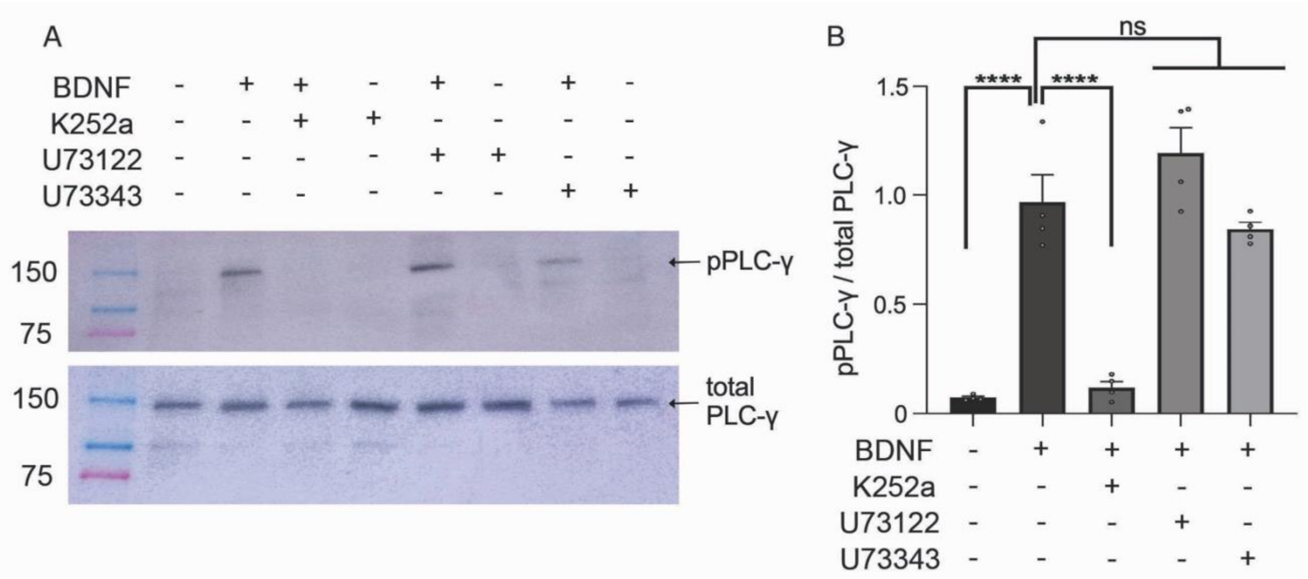

Supplementary Figure S1. K252a but not U73122 abolish BDNF-dependent phosphorylation of PLC-γ in mouse cortical neurons. (A) Upper panel, western blot analysis of the levels of phosphorylated PLC- γ (pPLC-γ) from lysates of DIV 7 neurons treated with BDNF, K252a (inhibitor of Trks activity) U73122 (inhibitor of PLC-γ activity**)**, U73343 (non-active structural analog of U73122), BDNF with K252a, BDNF with U73122 or BDNF with U73343. Lower panel, western blot analysis of the levels of total PLC-γ of the same treatment as upper panel. **(B)** Quantification of phosphorylated levels of PLC-γ (pPLC-γ) expressed as the ratio between the signal associated with phosphorylated PLC-γ (pPLC-γ) divided by the signal associated with total PLC-γ (pPLC-γ /PLC-γ). Neurons were not treated (control) or treated with BDNF (50 ng/ml) in the absence or presence of K252a, U73122 or U73343. n=4 independent experiments. The results are expressed as the mean ± SEM. ****p<0.0001. ns, non-significant. Statistical analysis was performed by one-way ANOVA followed by Bonferroni's post-test for multiple comparisons.

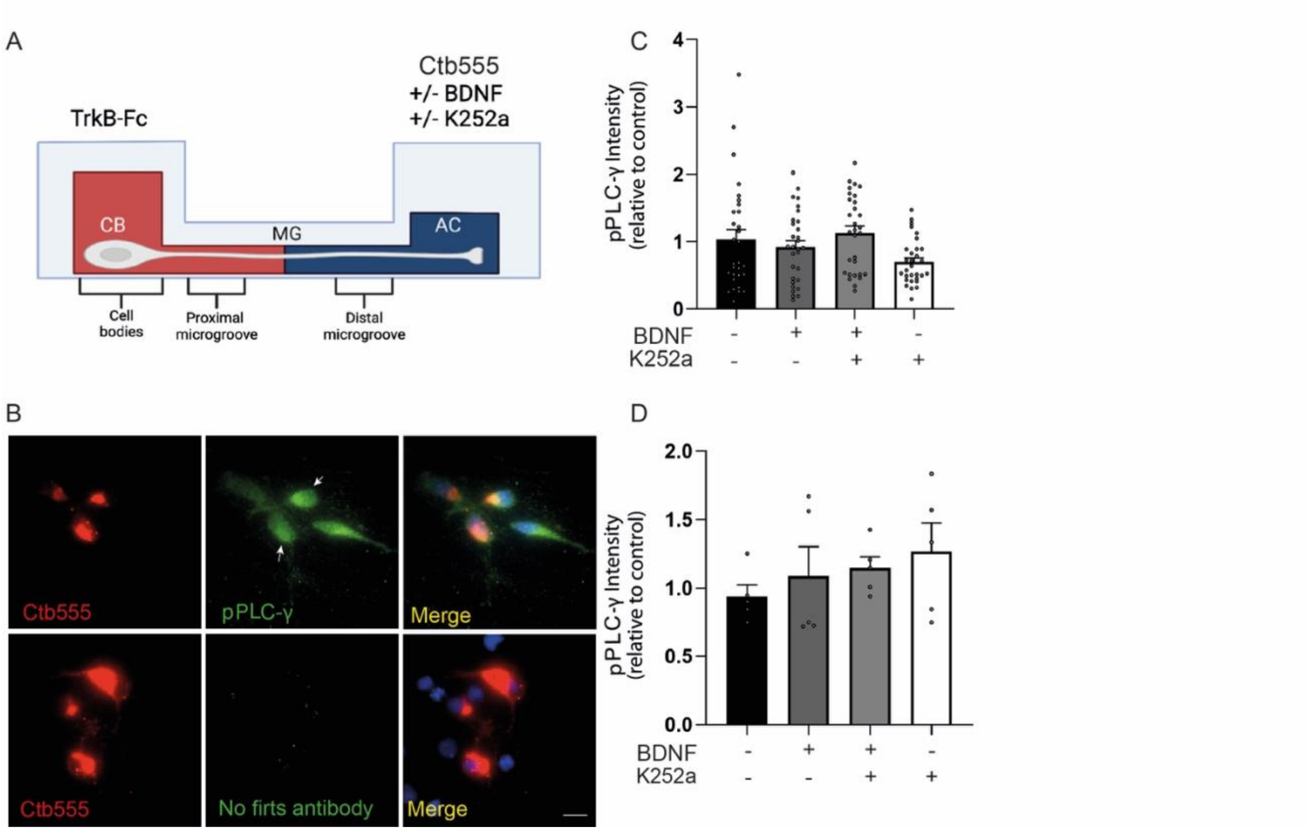

Supplementary Figure S2. Analysis of PLC-γ phosphorylation (pPLC-γ) in cell bodies and proximal axons of rat cortical neurons treated with axonal BDNF. **(A)** Schematic representation of the protocol used for stimulating neurons as indicated in Figure 2D. Axons were stimulated left alone (control) or stimulated with 50 ng/mL BDNF for 20 minutes (BDNF) or stimulated with BDNF in the presence of 0.2 μM K252a (BDNF + K252a). Axons were labelled with Ctb555 overnight before treatment to assess correct compartmentalization of the culture. CB, cell body compartment. MG, microgrooves. AC, axonal compartment. Brackets indicated the location used to quantify the fluorescence associated with pPLC-γ. Quantification of the fluorescence associated with pPLC-γ in distal microgrooves is presented in Figure 3. **(B)** Upper panels, representative images of Ctb488 (in red), phosphorylated PLC-γ (pPLC-γ, pY783.28, green) and Hoechst nuclear staining in blue in cell bodies of compartmentalized cortical neurons left unstimulated (control). Lower panels, cell bodies of compartmentalized cultures without primary antibody (control of pPLC-γ immunostaining). Scale bar, 10 µm. **(C)** Quantification of pPLC-γ in the somas of neurons retrogradely labeled with Ctb555. n=30 neurons from 3 independent experiments. **(D)** Quantification of pPLC-γ in axons proximal to cell body compartment. Quantification of the immunofluorescence signal associated with pPLC-γ was performed in a rectangular ROI drawn in the border between the beginning of the microgroove and the CB and continuing for 30 µm towards the microgroove that was delimited by Ctb555 fluorescence. n=5 chambers (the value in each chamber corresponds to the average of 5 different microgrooves) performed in five independent cultures. Statistical analysis was performed by one way ANOVA without significant differences.

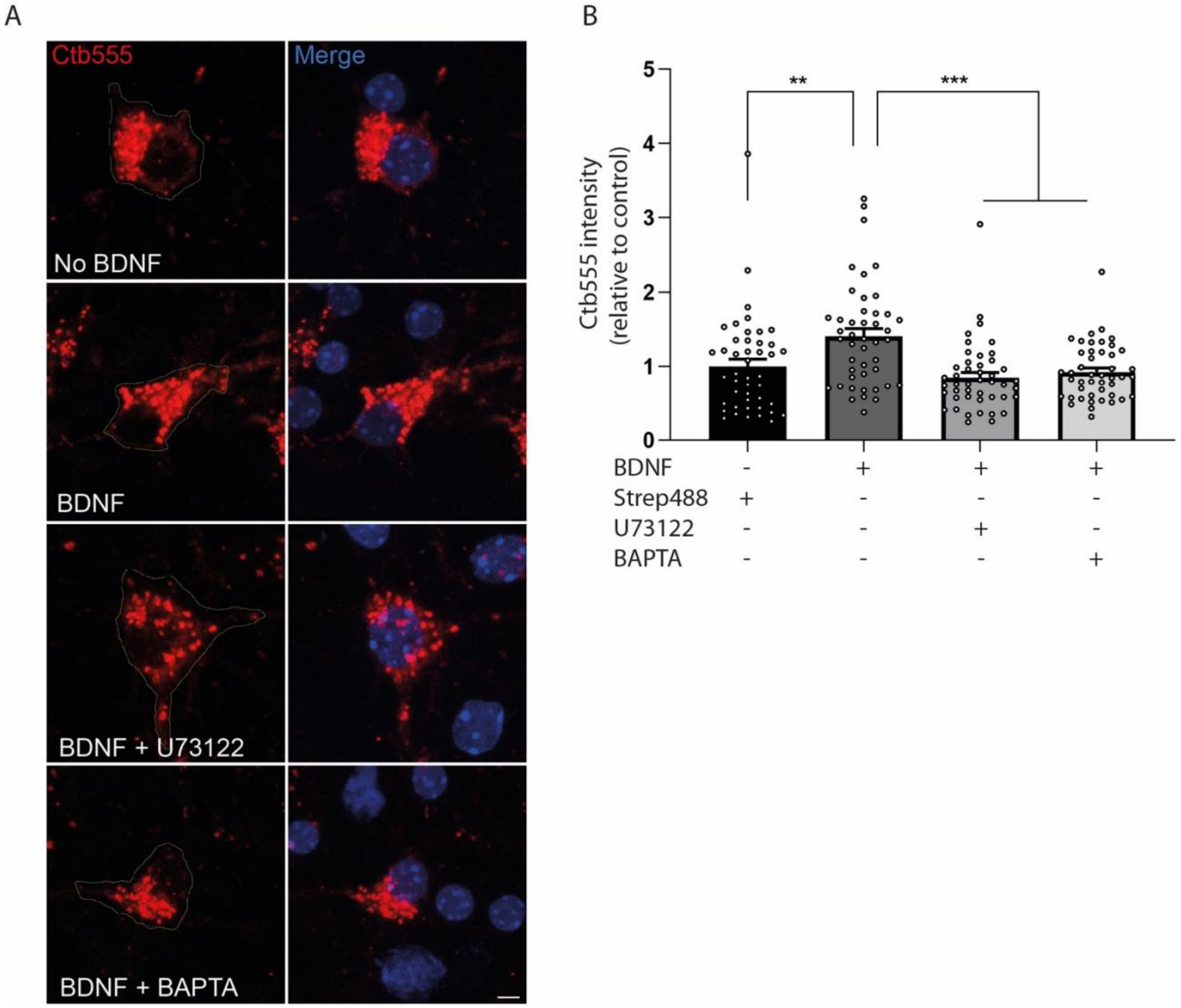

Supplementary Figure S3. BDNF in axons increases Ctb555 transport to neuronal cell bodies of compartmentalized mouse cortical neurons. (A) Representative images of neurons labelled with Ctb555 (in red) treated with DyLight 488-conjugated streptavidin alone (No BDNF, control condition) or with biotinylated BDNF coupled to DyLight 488-conjugated streptavidin (f-BDNF, 150 ng/mL) for 6 hours in the absence or presence of 5 µM U73122 (BDNF + U173122) or 20 mM BAPTA-AM (BDNF + BAPTA). Scale bar, 5 mm. **(B)** Quantification of fluorescent intensity associated with Ctb555 in cell bodies of neurons containing Ctb555. The values were expressed as relative to control. n= 45 neurons from 3 independent experiment. Statistical analysis was performed by one-way ANOVA followed by the Bonferroni correction for multiple comparisons. *** P=0.0004, ** P=0.0041.

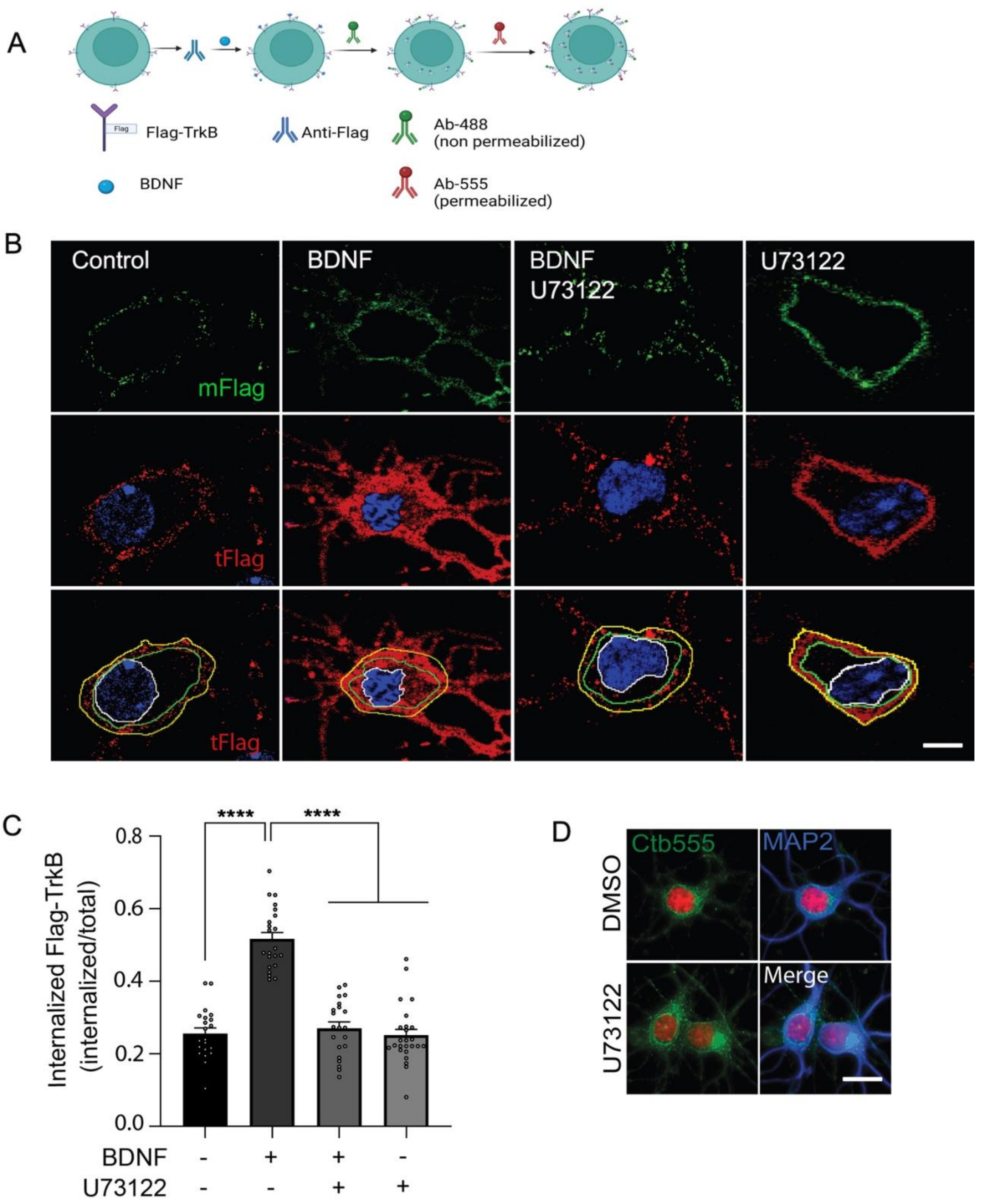
A
Flag
Flag-TrkB
Anti-Flag
Ab-488
(non permeabilized)
BDNF
Ab-555
(permeabilized)
B
Control
BDNF
BDNF
U73122
U73122
mFlag
tFlag
tFlag
C
****
****
Internalized Flag-TrkB
(internalized/total)
0.8
0.6
0.4
0.2
0.0
BDNF - + + -
U73122 - - + +
D
Ctb555
MAP2
DMSO
U73122
Merge

Supplementary Figure S4. PLC-γ activity is required for TrkB internalization in rat cortical neurons. (A) Schematic representation of the immunoendocytosis protocol used to study Flag-TrkB internalization. Neurons (DIV 6) were transfected with a plasmid expressing Flag-TrkB. After 48 hours, the neurons were incubated with an anti-Flag antibody. Then, the neurons were treated with BDNF (50 ng/mL) in the presence or absence of U73122 (5 µM) for 20 minutes at 37°C to induce endocytosis. Finally, the neurons were fixed, and the Flag epitope was detected by immunostaining. The plasma membrane-associated anti-Flag antibody was recognized with an anti-mouse Alexa 488 antibody (mFlag, green) before cell permeabilization; then, cells were permeabilized, and the total Flag epitope was recognized with an anti-mouse Alexa 555 antibody (tFlag, red). **(B)** Representative images of the endocytosis of Flag-TrkB in control cells treated with BDNF, U73122 or BDNF with U73122. The upper figure shows plasma membrane-associated Flag-TrkB (mFlag, in green), and the lower images show total Flag-TrkB in red (tFlag); nuclei labeled with Hoechst are shown in blue. The yellow line delimits the whole cell and the total Flag-associated fluorescence (red), the space between the green line (delimiting membrane-associated Flag-TrkB) and the white line (delimiting the nucleus) is the cytosol-associated Flag-TrkB. Scale bar, 5 µm. **(C)** Quantification of internalized Flag-TrkB was accomplished by dividing the fluorescence intensity associated with internalized Flag-TrkB (only the red fluorescence signal located between the green and white lines) by the fluorescence intensity associated with total intracellular Flag-TrkB under each treatment condition. n= 26-29 neurons from 3 independent experiments. The results are expressed as the mean ± SEM. ****$p < 0.01$. Statistical analysis was performed by one-way ANOVA followed by Bonferroni's post-test for multiple comparisons **(D)** Representative image of basal Ctb555 endocytosis in the presence or absence of U73122. No BDNF was added to the neuronal cell cultures. Green, Ctb555; blue, Map2; red, Hoechst. Scale bar, 10 µm.
